# Role of Traditional Chinese Medicine in Bone Regeneration and Osteoporosis

**DOI:** 10.3389/fbioe.2022.911326

**Published:** 2022-05-31

**Authors:** Zhicai Peng, Ronghua Xu, Qinjian You

**Affiliations:** Department of Orthopaedics, Yongchuan Hospita of Traditional Chinese Medicine, Chongqing Medical Uninersity, Chongqing, China

**Keywords:** osteoporosis, bone regeneration, traditional Chinese medicines, decoction, adrenocorticotropic hormone (ACTH)

## Abstract

According to World Health Organization (WHO), osteoporosis is a systematic bone disability marked by reduced bone mass and microarchitectural degeneration of osseous cells, which leads to increased bones feebleness and fractures vulnerability. It is a polygenetic, physiological bone deformity that frequently leads to osteoporotic fractures and raises the risk of fractures in minimal trauma. Additionally, the molecular changes that cause osteoporosis are linked to decreased fracture repair and delayed bone regeneration. Bones have the ability to regenerate as part of the healing mechanism after an accident or trauma, including musculoskeletal growth and ongoing remodeling throughout adulthood. The principal treatment approaches for bone loss illnesses, such as osteoporosis, are hormone replacement therapy (HRT) and bisphosphonates. In this review, we searched literature regarding the Traditional Chinese medicines (TCM) in osteoporosis and bone regeneration. The literature results are summarized in this review for osteoporosis and bone regeneration. Traditional Chinese medicines (TCM) have grown in popularity as a result of its success in curing ailments while causing minimal adverse effects. Natural Chinese medicine has already been utilized to cure various types of orthopedic illnesses, notably osteoporosis, bone fractures and rheumatism with great success. TCM is a discipline of conventional remedy that encompasses herbal medication, massage (tui na), acupuncture, food, and exercise (qigong) therapy. It is based on more than 2,500 years of Chinese healthcare profession. This article serves as a comprehensive review summarizing the osteoporosis, bone regeneration and the traditional Chinese medicines used since ancient times for the management of osteoporosis and bone regeneration.

## Introduction

According to WHO, by 2022, the number of persons living with osteoporosis in the America is projected to boost from around 10 million to over 14 million (Bartl and Frisch, 2004; Burge et al., 2007). Due to modern civilizations’ constantly expanding life expectancy, the global implications of osteoporosis and delayed bone repair are massive. As a result, the clinical need to reverse bone loss, promote bone development, and enhance osteogenesis is rising, and it is a critical problem for medical professionals (Russow et al., 2019). Hormonal and metabolic abnormalities are the most common etiologies of osteoporosis. Nevertheless, osteoimmunology has shown that the immune cells and immunological parameters perform key regulating roles in the progression of osteoporosis. The immune system’s aberrant stimulation disrupts the equilibrium between osteoclasts and osteoblasts, creating instability in bone remodeling and osteoporosis (Guerrini and Takayanagi, 2014; Limmer and Wirtz, 2017). Over 50 years of age, about one-third of women and one-fifth of men will develop at least one bone fracture due to osteoporosis (Sözen et al., 2017).

For the prophylaxis and therapy of osteoporosis, the Federal Drug Administration (FDA) of United States has authorized a number of medications that function by preventing bone resorption or reduce the incidence of fractures (anabolic agents). Bisphosphonates, competitive oestrogen receptor modulators, and the monoclonal antibody denosumab are examples of drugs that prevent bone degradation but do not induce bone regeneration (Russow et al., 2019). Abaloparatide and Teriparatide are the currently US Food and Drug Administration (FDA) approved anabolic agents for the treatment of osteoporosis in the United States approved in 2017 and 2002 respectively (Miller et al., 2016; Haas and LeBoff, 2018). The principal treatment approaches for bone loss illnesses, such as osteoporosis, are hormone replacement therapy (HRT) and bisphosphonates. Continuous HRT is associated to high risk of mammary cancer and endometrial, as well as coronary artery problems and other cardiac disorders whereas bisphosphonates are responsible for the osteonecrosis of the jaws and bones (Dinger et al., 2016; He et al., 2017; Spivakovsky, 2017). The clinical usage of HRT and bisphosphonates is limited due to these adverse effects. As a result, new treatment strategies are needed to produce osteoporosis treatments that are somewhat liable to cause fewer adverse reactions (Lungu et al., 2018). Cathepsin-K is a cysteine protease that is found in significant amounts in osteoclasts. Even though there are various additional cathepsins with large anatomical concentrations, any antagonists for clinical usage must have a higher selectivity if they are to be safe. Balicatib preceded through phase 2 clinical studies, however due to cutaneous and respiratory adverse effects, onward research was halted (Reid, 2008). Odanacatib concluded effectively phase 2 studies with no apparent potential risks. It decreases osteoclastogenesis indicators by around 70% and induces variations in spine bone concentrations that are similar in many aspects with active bisphosphonates if administered as a fortnightly oral route (Bone et al., 2007).

Traditional Chinese medicines (TCM) have grown in popularity as a result of its success in curing ailments while causing minimal adverse affects. Natural Chinese medicine has already been utilized to cure various types of orthopaedic illnesses, notably osteoporosis, bone fractures and rheumatism with great success (Mukwaya et al., 2014; Suvarna et al., 2018). The widespread use of TCM for musculoskeletal disorders has been existed for many years, both in rural as well as in urban regions. Topical herbal formulations are especially beneficial in cases of joint injuries, inflammations, but potentially in fractured bones (Jiang and Ye, 2005). According to latest research findings, these Chinese medicinal approaches for the management of osteoporosis emerge to have equally anticatabolic and anabolic impacts by boosting osteogenesis and minimizing extremely unbalanced bone turnover, resulting in improved bone mineral density as well as minimal bone microstructural degradation (Wang et al., 2017a; He et al., 2017).

This review focuses on and acknowledges the scientific proof for the possible usage of traditional Chinese medicine in the management of osteoporosis and bone regeneration.

## Osteoporosis and Bone Regeneration

Osteoporosis is a skeletal disorder that is characterized by poor bone microarchitecture/mineralization, reduced bones’ mineral density (BMD), and/or diminished bone growth. This asymptomatic illness typically goes untreated till it emerges as minimal fractures of the spine, wrist, pelvis, proximal humerus, and/or hips, which commonly necessitates hospitalizations (Cosman et al., 2014; Jeremiah et al., 2015). There are two types of osteoporosis: primary and secondary. Primary osteoporosis: 1) postmenopausal affects mostly the vertebral column trabeculae and is common in women aged 45 to 65. It is caused by the oestrogen insufficiency and impairment of ovarian function. 2) senile in women over the age of 70–75, which is caused by a reduction in vitamin D and calcium consumption, decreased vitamin D generation and metabolism in the body, diminished intestinal absorption, and the ageing process: 3) Idiopathic (juvenile) fractures—a rare type that affects people of reproductive age (below 40). Disorders or medicines that damage bone cells cause secondary osteoporosis. Continuous utilization of selective serotonin receptor inhibitors, glucocorticoids, androgen deprivation therapy, proton pump inhibitors, thiazolidinedione, calcineurin inhibitors, heparin, and various chemotherapies, such as methotrexate, might cause osteoporosis (Ivanova et al., 2015).

Primary osteoporosis is frequently linked to advanced age and a lack of sex hormones. The ongoing degradation of the trabeculae in bone causes age-related osteoporosis. Furthermore, the decrease in oestrogen levels in older women leads to greater osteoporosis. Sex-hormone–binding globulin downregulates the male sex hormones as they mature, which might also result in bone loss over time (Jeremiah et al., 2015; Society, 2021). Multiple concomitant conditions and/or drugs can lead to secondary osteoporosis. The imbalance of vitamin D, calcium, and sex hormones is frequently linked in disorders associated with osteoporosis. Men on androgen-deprivation therapy (ADT) for prostate cancer, for example, have a higher likelihood of osteoporosis (Shahinian et al., 2005; Schnatz et al., 2011). Excessive glucocorticoid synthesis in Cushing’s syndrome has now been reported to hasten osteoporosis (Kawamata et al., 2008). Furthermore, several inflammatory disorders, including rheumatoid arthritis, may necessitate long-term glucocorticoid medication, which has now been linked to secondary osteoporosis. Glucocorticoids, in particular, are thought to be even more prevalent drugs associated to drug-induced osteoporosis (Buckley et al., 2017). Reduced blood supply in radiotherapy and chemotherapeutic therapies focused resistant cancerous cells *via* modifying the bones microenvironment as well as inhibiting them from establishing a quiescent state. Such results indicate that limiting radiation- and chemotherapy-induced pericyte growth *via* blood supply manipulation may augment existing anticancer therapeutic strategies for managing or preventing bone metastases (Singh et al., 2019).

Bones have the ability to regenerate as part of the healing mechanism after an accident or trauma, including musculoskeletal growth and ongoing remodeling throughout adulthood. In order to maximize osseous fixing and re-establish skeletal function, bone rehabilitation is composed of a very well sequence of biochemical pathways of bone initiation and conduction, comprising a set of cell types and extracellular and intracellular molecular-signaling pathways, with a distinguishable spatial and temporal sequence (Einhorn, 1998; Cho et al., 2002; Bates et al., 2018). The much more prevalent form of osteogenesis in the clinical context is fracture repair, which mimics the typical embryonic osteogenic cascade, comprising of endochondral and intramembranous bone formation. Almost all of the orthopaedic traumas cure without the development of scar tissue, and bone regenerates like its pre-existing qualities completely recovered, with the freshly formed bones finally unidentifiable from the nearby healthy bone (Ferguson et al., 1999; Dimitriou et al., 2011). Conversely, there are examples where bone regrowth is hindered during bone remodeling, about 13% of tibia fractures being linked with fracture non-union or disorder union. Furthermore, there are other situations in oral and maxillofacial and orthopaedic surgical procedure where substantial quantities of bone healing are needed, including bony restoration of large bone defects caused by tumour resection, trauma, bone deformities, and infection, or situations wherein the regenerative procedure is impaired, like osteoporosis and a vascular necrosis (Audigé et al., 2005).

Presently there are a variety of medical procedures in the surgeon’s therapeutic strategies for all of the mentioned scenarios wherein the natural process of bone healing is either damaged or simply ineffective, that could be employed by itself or in conjunction for the augmentation or management of these complicated clinical situations, which can frequently be resistive to therapy, constituting a medical and economical challenge. Distraction osteogenesis and bones transfer are two common techniques used in clinical practice to accelerate or enhance bone healing. A variety of bone-grafting techniques, including allografts, autologous bone grafts, growth factors, and bone-graft replacements are also used (Aronson, 1997; Giannoudis et al., 2005; Giannoudis and Einhorn, 2009).

## Traditional Chinese Medicines in Osteoporosis and Bone Regeneration

Since ancient times, Traditional Chinese medicines (TCM) were used in hospitals and private clinics to cure skeletal disorders, and it is aneconomical substitute to commercialized pharmaceutical medicines. TCM are used in more than 130 countries across the globe. It is assumed that the application of TCM on damage tissues effect directly on the traumatized organ and trigger improvements and bone healing. Traditional Chinese Medicine’s extensive use for musculoskeletal disorders has endured the test of time in both rural and urban settings. In joint sprains, inflammatory disorders, and even fractured bones, topical applications of natural remedies are useful (Hsiao, 2007; Mukwaya et al., 2014). TCM has already been utilized for the management of musculoskeletal disorders, particularly bone fractures, osteoporosis, and rheumatism with great success (Mukwaya et al., 2014). TCM evolved from mythological therapy into a herbal medicine system. The impacts of several Chinese medicines were examined and vetted over many years of clinical practice. TCM differs greatly from modern medicine in terms of practice and theory (Wong and Rabie, 2006). TCM was added in the 11th edition of the International Statistical Classification of Diseases and Related Health Problems by the World Health Organization (WHO) in 2018 (Organization, 2004).

**FIGURE 1:**
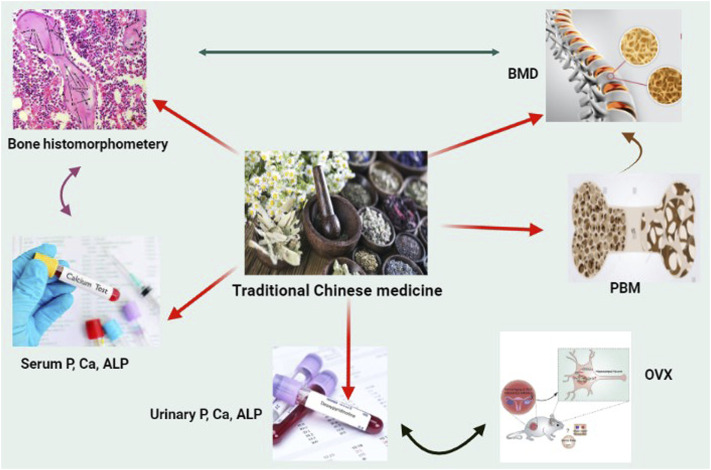
Natural Chinese medicine’s therapeutic promise in the treatment of osteoporosis. When faced with the obstacles of oestrogen or androgen shortage, excessive hormone medicines, and weightlessness, bone quality will be severely harmed. Although various natural Chinese medications may be good choice for improving skeleton growth and preventing bone loss.

TCM is a discipline of conventional remedy that encompasses herbal medication, massage (tui na), acupuncture, food, and exercise (qigong) therapy. All these approaches are equally practiced, in this review, however we will be more focusing on herbal medication component of the Traditional Chinese Medicine. It is based on more than 2,500 years of Chinese healthcare profession (Sheng et al., 2012). The zàng-fu theory is among the fundamental concepts of TCM. The terminology zàng focuses on five key components, which include the liver, heart, lung, kidney and spleen, which include the stomach, gallbladder, small intestine, urine bladder, large intestine, and Sānjiaō. The kidneys are some of them, and it is thought to be associated to skeletons. In contrast to the Modern health conception of kidneys, the TCM idea of kidneys seems more like a method of characterizing a group of interconnected components than a physiological organ. The kidney’s primary duties are to build bones, control human growth and development, and generate marrow to fill the brain. Numerous kidney-nourishing herbal medications can restore bones, as per the Chinese medicine kidney theory, and are thus prescribed to cure bone-related disorders including osteoporosis. One of these medicines’ mode of action might increase osteoblastogenesis (Liu, 2018; Matuk, 2006). According to published research findings, such traditional Chinese medicines for the therapy of osteoporosis seem to have both anabolic and anticatabolic impacts besides boosting osteogenesis and minimising unbalance osteoclast activity, resulting in enhanced bone mineral density and biomechanical properties, as well as diminished bone microstructural degeneration (Figure 1) (He et al., 2017; Suvarna et al., 2018).

Bone regeneration is a sophisticated biochemical procedure that starts with localized hemorrhage and inflammation, and then proceeds to the production of cartilage, soft extracellular matrix tissue, and fresh bone *via* the complex actions of mesenchymal progenitor cells. TCM emphasizes on the total influence of the patient’s body on the bone lesions in healing musculoskeletal disorders, rather than just the local therapy as is usual in western treatment. Therefore, TCM uses both interior therapies in conjunction to external therapy. TCM considers 3 phases when it comes to bone deformities: 1) during first phase, there is harm to the blood vessels due to stagnation of “qi” and blood, and also obstructed meninges, which together leads to pain; 2) in the second phase, the inflammation eventually subsides and the pain is significantly reduced, but the bruises remain, and connective tissues are not repaired; 3) in the third phase, the overall bone damage has now been healed, but remedy to pacify and improve bone regeneration, and improve the internal environment is yet required (Tang et al., 2021; Zhang et al., 2021). Icariin (ICA) being a traditional Chinese medicine which was found to repair the vascular system and successfully modulate intracellular senescence in bones. Furthermore, it is unknown if ICA can impact endothelial cells’ angiogenic capacity through changing apoptosis (Li et al., 2022). Traditional Chinese medicines having bone regeneration and antiosteoporosis activity are summarized in Table 1.

**TABLE 1:** Summary and mechanism of Traditional Chinese medicines (TCM) used for bone regeneration and antiosteoporosis.

TCM	Extract/compounds	Study type	Effect on bones	References
*Psoralea corylifolia*	Extract	*In vivo*	Increase bone calcification and hyperosteoidosis	Miura et al. (1996)
*Gynochthodes officinalis*	Extract	*In vivo*, *In vitro*	↑Bone Mineral Density, ↑Bone Mineral Content, ↑serum P, Ca^2+^	(Seo et al., 2005; Li et al., 2009; Li et al., 2014a)
			Increases tibia ↑Bone Mineral Density, ↑osteoblasts, ↓osteoclast	
*Eucommia ulmoides*	Cortex extract	*In vivo*, *In vitro*	↑Bone Mineral Density, ↑bone microarchitecture	
*Curculigo orchioides*	Extract	*In vivo*	↑Bone Mineral Density	(Wong et al., 2007; Cao et al., 2008)
			↓bone fractures	
*Cuscuta chinensis*	Kaempferol	*In vivo*	↑Bone Mineral Density, ↑Bone microarchitecture and biomechanical parameters	Li et al. (2013)
*Epimedium brevicornum*	Icariin flavonoids	*In vivo*, *In vitro*	↑Bone Mineral Density, increases serum ALP, micro-architecture and biomechanical parameters	(Zhang et al., 2006; Liang et al., 2012)
Herba epimedii	Icarrin, icaritin	*In vivo*	improve osteoblast’sdifferentiation and proliferation	Vickers, (2017)
*Fructus ligustrum lucidum*	ligustroflavone, Specnuezhenide, salidroside	*In vivo*, *In vitro*	Activate osteoblast proliferation and bone development by runx2and BMP2 activation	(Wang et al., 2017b; Tang et al., 2018)
*Rhizoma drynariae*	Total flavonoids	*In vivo*	Augment osteoblast function through BMP2/Smad cascade	Chen et al. (2011)
*Puerariae radix*	Puerariae radixextract	*In vivo*	Increases osteoblast formation	Huh et al. (2006)
*Ecliptae herba*	Wedelolactone	*In vivo*, *In vitro*	Stimulate osteoblast differentiation and bone formation	(Lee et al., 2009; Liu et al., 2014a)
*Astragalus membranaceus*	Extract	*In vivo*	Stimulate fresh bone development on periodontal defects	Yang et al. (2016)
*Acer nikoense Maxim*	Acerogenin A	*In vitro*	↑(BMP-2, BMP-7, BMP-4), Stimulate osteoblast	Kihara et al. (2011)
*Caragana sinica Rhed*	Kobophenol A	*In vitro*	↓(NO-induced necrosis); regulate NF-κΒ, AP-1 and JNK signaling pathways, Augment osteoblast function	Lee et al. (2011a)
*Picrorrhiza kurrooa* Royle ex Benth	Apocynin	*In vitro*	↓(IL-6, ROS and TNF-α) Stimulate osteoblast	Lee and Choi, (2011a)
*Magnolia officinalis*	Honokiol	*In vitro*	↓(IL-6, TNF-α)	Choi, (2011)
			↑Bone Mineral Density	
*Sambucus williamsii Hance*	Vanillic acid	*In vitro*	↑(MAPK (MEK/ERK) mediated signaling pathway, ↑Bone microarchitecture and biomechanical parameters	Xiao et al. (2014)
*Salvia miltiorrhiza*	Salvianolic acid B	*In vitro*	↑ERK signaling pathway, ↑Bone Mineral Density, ↑Bone microarchitecture	Xu et al. (2014)
*Radix Ophiopogon japonicas*	Ophiopogonin D	*In vitro*	↓ROS; the FoxO3a-β-catenin signaling pathway, ↑Bone Mineral Density	Huang et al. (2015)
*Drynaria rhizome*	Neoeriocitrin, Naringin	*In vitro*	↑(Runx2, ALP)	Li et al. (2011)
			↑Bone Mineral Density, ↑Bone microarchitecture and biomechanical parameters	
*Poncirus trifoliate*	Poncirin	*In vitro*	↓(C/EBP-β, PPAR-γ) Increase bone calcification and hyperosteoidosis	Yoon et al. (2011)
*Helminthostachys zeylanica*	Ugonin K	*In vitro*	↑p38 MAPK- and ERK-mediated pathway, ↑Bone Mineral Density	Lee et al. (2011b)
Fruits and vegetables	Quercetin	*In vitro*	↑(ALP, Osx, Runx2) Increase bone calcification and hyperosteoidosis	Srivastava et al. (2013)
Ginseng	Ginsenoside-Rb2	*In vitro*	↑(ALP)	Huang et al. (2014)
			↓(IL-6, ROS)	
			↑Bone Mineral Density, ↑Bone microarchitecture and biomechanical parameters	
*Saussurea lappa*	Costunolide	*In vitro*	↑PI3K, JNK, PKC, ERK, p38	Lee and Choi, (2011b)
			↑Bone Mineral Density, ↑Bone microarchitecture and biomechanical parameters	
The genus *Rhamnus*	Emodin	*In vitro*	↑(BMP-2, PI3K, Akt and MAPK) pathways	Lee et al. (2008)
			↑Bone Mineral Density, ↑Bone microarchitecture	
*Cnidium monnieri*	Imperatorin	*In vitro*	↑(bone nodule, BMP-2, phosphorylation of SMAD 1/5/8); ERK and p38 -dependent pathway	Tang et al. (2008)
			↑Bone Mineral Density, ↑Bone microarchitecture	
*Sophora japonica*	Genistein	*In vitro*	provoke ERα regulation via MAPK/NF-κB/AP-1 pathway	Liao et al. (2014)
			↑Bone Mineral Density	
*Rhodiola rosea*	Salidroside	*In vitro*	↑(BMP-2, BMP-7, phosphorylation of ERK1/2and SMAD 1/5/8) ↑Bone Mineral Density	Chen et al. (2013)
*Herba siegesbeckia*	Kirenol	*In vitro*	↑(BMP-2, β-catenin); BMP and Wnt/β-catenin pathways	Kim et al. (2014)
Bu-Shen-Tong-Luo Decoction	Combination of three ancient Chinese formulae	*In vivo*	↓bone resorption and ↑angiogenesis	Yuan et al. (2018)

### Herba Epimedii

The dried aerial component of *Epimedium* species like *Epimedium sagittatum* (Yin Yang Huo) is known as Herba Epimedii (HE). HE is among the best commonly recommended herbs in Chinese osteoporosis formulations (Qin et al., 2005). The plant is harvested, stripped of stiff stems and unwanted particles, and shade or Sun drying is done in the autumn and summer. Sichuan, Shaanxi, Guangxi, Liaoning, and Hubei states yield the majority of it. Flavonoids, phenolic glycosides, ligans, penethylol glycosides, ionones and sesquiterpenes have all been extracted from Epimedium species. In an aging or ovariecto-mized (OVX) rat model, HE as a solo herb or even as a herb combination could prevent bone resorption (Zhu, 1998; Wong and Rabie, 2006). According to Gao, *in vitro* production of the chick embryo femur was stimulated by Epimedium in injectable formulation (Gao, 1985). HE extract has been shown in clinical trials to inhibit bone resorption and enhance E2 and osteocalcin concentrations. In OVX rats, the total flavonoid fraction of HEP started to improve BMD, increased E2 levels, and lowered systemic IL-6 levels (Jiang et al., 2002). Icariin, a flavonoid biomarker in HE, is thought to be the main bioactive element responsible for its bone-regeneration properties (Wong and Zhang, 2013). In an experiment conducted, OVX C57BL/6 test mice were injected icariin at a dose of 0.3 mg/g day for 6 weeks to determine the *in vivo* bones protecting properties of the compound. Icariin was reported to prevent the degradation of bone density and integrity in the femur bone (Mok et al., 2010).

Bone and bone marrow vascular are essential for supply of nutrients, oxygen, and secrete angiocrine factors essential for maintenance, survival, and self-renewal of progenitor and stem cells. Vasculature produces nurturing slots for blood-forming stem cells and bones in the skeletal system. Hematopoiesis is regulated by intact blood vessels and also aid in the formation of bones during repair, development, and re-generation. Malfunctioning of vasculature induces bone diseases, skeletal aging, and blood disorders (Chen et al., 2020). Physical activity can improve blood supply and vascularization to an organ, however, the curative/preventive potentials of physical activity on bone repair due to contribution of vascularization due to such activities have not been investigated specifically (Wazzani et al., 2021). The two major components Icaritin and Icariin of *Epimedium sagittatum* (Yin Yang Huo) also known as Herba Epimedii (HE) have been shown to have prominent effects in vascularization and bone formation. Icaritin reduced the incidence of steroid-associated osteonecrosis and inhibited intravascular thrombosis and extravascular lipid-deposition upon oral administration to rabbits (Zhang et al., 2009a). Similarly, an icariin/tricalcium phosphate porous scaffold enhanced new vascular and bone formation in a rabbit model of femoral head osteonecrosis after 12 weeks of treatment (Xie et al., 2015).

By methodologies of cell culture *in vitro* and detection of 3H-TdR integrating DNA of bone marrow cells, Liu et al. investigated the impact of Epimedium sagittatum polysaccharides on DNA replication of bone marrow cells of “yang deficiency” animal model produced by hydroxyurea. The findings demonstrated that following 100 μg dose of *Epimedium sagittatum* polysaccharides were applied, cell multiplication improved by 72% and DNA replication enhanced by 68 percent (Liu et al., 1991). Yu et al. investigated the effects of the Chinese herb *Guizhou epimedium* on osteoporosis and osteoclastic bone resorption. In order to evaluate the activity of the Chinese herb *in vitro*, osteoclasts were extracted and cultivated. The *in vivo* activity of this herb had also been studied in rats that had osteoporosis caused by ovariectomy, and the findings were correlated to the impact of estradiol in the similar group of rats. The epimedium was found to decrease osteoclastic degradation of bones in an *in vitro* research. Apparently the estradiol and epimedium proved to boost up mineral content and accelerate bone regeneration in the *in vivo* study (Yu et al., 1999).

### Fortune’s Drynaria Rhizome (Rhizoma Drynariae)

The dried rhizome of the perennial Drynaria fortunei is known as Gu Sui Bu. It has been assumed to be an effective bones healer in traditional Chinese medicine. All year long, the rhizome can be assembled and processed. Guangdong, Zhejiang, Sichuan Hubei, and Guangxi provinces yield the Gu Sui Bu (Xu et al., 1991). Drynaria fortunei is a perennial herb that is often used to treat musculoskeletal diseases. Drynaria fortunei also possesses anti-osteoporosis properties. Drynaria fortunei’s anti-osteoporosis activity was also supported by the finding in rats that it inhibited ovariectomy-induced bone resorption thus maintained the fine particle surfaces of the trabeculae (Anderson et al., 2002). In an *in vitro* model, Drynaria fortunei extract was also demonstrated to have a powerful blocking action on cathepsin-K induced decomposition of collagen via reducing cathepsin-K activities. Furthermore, naringin is a flavonoid with estrogenic action that is found naturally in Drynaria fortune as bioactive component. Naringin when administered orally, increases BMP-2 expression, inhibits retinoic acid induced osteoporosis in rodents, enhances the growth and osteoblast development of bone mesenchymal stem cells, and induces osteogenesis through stimulating oestrogen receptor phosphorylation in osteocytes (Jeong et al., 2005; Wei et al., 2007; Dai et al., 2009).

Lin and his colleagues used an *in vitro* bone cell culture to look into the biochemical impact of ten various traditional Chinese medicines. The research model focused on the rat osteoblast-osteoclast co-culture technique. Different testing compounds were introduced when the cells had grown to 80% confluences. A colorimetric technique was used to assess the mitochondrial actions of osteocytes, following exposure to several Chinese medicine treatments. To assess bone cell activity, biochemical indicators like lactate dehydrogenase (LDH), protein content, acid phosphatase (ACP) titer, and alkaline phosphatase (ALP) were analysed. Just 4 out of 10 Chinese herbs had possible good results on bone cell culture when cultured for 24 h, and only Drynaria fortunei showed ubiquitous significant positive impact on bone cell metabolism. Drynaria fortunei’s main positive activity on bone cells is thought to be due to the triggering of apoptosis in the osteoclast cell (Lin et al., 2002).

### Curculigo orchioides Gaertn

*Curculigo* plants have been reported in over 20 different species around the globe. They are indigenous to South America, Africa, Asia and Oceania’s tropical and subtropical climates. *Curculigo orchioides* is a kind of *curculigo*. In Chinese medicine, *Curculigo orchioides Gaertn* (COG, “Xian Mao”), it was applied topically for the management of knee and spine joints arthritis, leg fatigue and diarrhoea. COG seems to have antitumor effects and antioxidant, and could be employed as an antiosteoporotic herbal agent, according to latest research (Ramchandani et al., 2014; Vickers, 2017; Hejazi et al., 2018; Cui et al., 2019). COG has been studied *in vivo* for its antiosteoporotic activities in TCM for the management of postmenopausal women with osteoporosis. COG reduced trabecular bone degradation in ovariectomized rats’ tibias by reducing osteoclast activity and boosting serum calcium, phosphorus and OPG levels while having no effect on body or uterus size. After COG dosing, serum concentrations of bones degradation associated makers like corticosterone, TRAcP, DPD/Cr, and ACTH were reduced (Cao et al., 2008). COG might even stimulate bone regeneration after healing process, according to Wong et al. COG’s principal biologically active component, curculigoside (CCG; phenolic glycoside), is reported to have antiosteoclastic and osteogenic properties (Wong et al., 2007; Liu et al., 2014b).

Curculigoside upregulated the osteogenic action in human amniotic fluid derived stem cells in a dose-dependent way, according to a latest *in vitro* analysis, which revealed that CCG up-regulated osteogenic action in a dose-dependent way, such as upregulation of Collagen I and osteopontin, increased the ALP action, and calcium accumulation. The observation that Cyclin-D1 and β-catenin are both upregulated at the same time suggests that such actions are regulated through the β-catenin/Wnt signaling cascade (Liu et al., 2014b). By modulating osteogenic growth, differentiation, and proinflammatory cytokine concentrations, CCG has indeed been demonstrated to prevent rat calvarial osteoblasts from dexamethasone (DXM)-induced toxicity. The consequences of DXM on the concentrations of osteoblast development biomarkers such as OPG, ALP and β-catenin were reverted, suggesting that CCG could be a good candidate for the management of postmenopausal osteoporosis (Zhu et al., 2015). Phytochemicals extracted from COG have been demonstrated to exhibit antiosteoporotic effects *in vitro* and in rat calvarial investigations, such as the encouragement of osteoblast differentiation and proliferation, enhanced bone forming activities, and suppression of osteoclastogenesis (Jiao et al., 2009; Wang et al., 2017c). In summary, *C. orchioides’* induction of osteogenesis may be a good impact resulting in reduction of osteoporosis, and the medicine’s suppression of ROS is a valuable quality. Furthermore, several COG-derived compounds have been shown to inhibit osteoclastic demineralization. More exploration is needed to establish the therapeutic constituents of COG, molecular pathways, and signalling routes as well as the biochemical basis of osteoporosis.

### Fructus Ligustri Lucidi

Fructus Ligustri Lucidi (FL) is most often used to purify the kidneys and augment the bones as a singular herb. In ovariectomized rats which were fed whether with a regular 0.6% Ca dietor a reduced 0.1% Ca diet, orally, FL extract enhanced bone density and bone’s mechanical integrity at the diaphysis of femurs, tibias and lumbar vertebrae. Additionally, FL modulates calcium equilibrium in older women by modifying the parathormone’s vitamin-D axis and increasing absorption of calcium *in vivo*, implying that FL might be an appropriate therapy for restoring calcium and vitamin-D equilibrium (Haung and Yang, 2003; Zhang et al., 2008a; Zhang et al., 2008b). Even though oleanolic acid, the significant bioactive compound in FL, had enhanced the osteoblastic delineation of osseous mesenchymal stem cells and osteoprotective activity in ovariectomy-induced osteoporotic rats *in vitro*, more research is needed to determine whether this chemical contributes to the regulatory oversight of FL on calcium equllibrium (Haung and Yang, 2003).

### Er-Xian Decoction

Er-Xian decoction (EX) is a multi-herb composition consisting of six different herbs: *Cortex phellodendri, Radix morindae officinalis, H. epimedii, Rhizome curculiginis, Radix angelicae sinensis and Rhizome anemarrhenae*. It has proven effective in reducing osteoporosis, perimenopausal syndrome, and ageing problems in older patients. It is therapeutically useful in alleviating menopausal symptoms by boosting the level of circulating estradiol. EX alleviated the menopausal symptoms by increasing antioxidant and endocrine activity, through the stimulation of catalase (CAT) and aromatase detoxification channels (Liu et al., 2005; Sze et al., 2009). EX has been proven to have anti-osteoporotic actions comparable to estrogens and has attributed enormously to the prophylaxis or therapy of bone loss caused by ovariectomy in rats. It has beneficial benefits on bone while having just minimal impact on the uterus, demonstrating that EX is acceptable to be used for bone health management (Nian et al., 2006).

### Eucommia Ulmoides Oliv

Eucommia Ulmoides Oliv (EO) is the extract of leaf and cortex that has reported to inhibit osteolysis and bones density depletion, as well as stimulate osteogenesis. Ha and his co-researchers investigated the phytochemicals in a portion of extract of *Eucommiae cortex* and found that these constituents play active role in every sequence of the pathways for initiating osteoblast to expedite osteogenesis and impeding osteoclast action to block osteolysis by evaluating on osteoporosis the clinical efficiency of these crude extract, like methanol, ethyl acetate, chloroform, aqueous, and butanol fractions. The oral administration of EO over a 4-month time frame in adult OVX rats was found to prohibit the degradation of trabecular micro-architecture and loss of estrogen deficiency-induced osseous micronutrients, thus sustaining physiological fluency of the bone. Moreover, EO juice was reported to activate the secretion of GH, controlling bone growth, development and bone formation, implying that EO could be a promising candidate for the treatment of osteoporosis (Ha et al., 2003; Zhang et al., 2009b).

### Fructus Psoraleae (Bu Gu Zhi)

The dehydrated mature fruits of the perennial herb *Psoralea corylifolia* L. are known as Bu Gu Zhi. It belongs to family Leguminosae. The plant is harvested and processed in the autumn. After that, the fruit is scraped clean and free of extraneous debris. Anhui, Shanxi, Henan, and Sichuan states yield the majority of the plant. Healthy rats were given the acetone seed extract (the n-hexane ethyl acetate elution) of P. corylifolia, which resulted in a considerable increase in bones mineralization and serum inorganic phosphorus. Bone regeneration and serum levels of phosphorus were elevated in rachitic rats (already nourished with vitamin D free diet) when n-hexane elution were orally administered. In rachitic rats, administration of n-hexane elution showed a significant reduction in osteoid quantity and an enhancement in hyperosteoides. These findings indicate that n-hexane elution could be effective treatments for osteomalacia, osteoporosis, broken bone, and other skeletal disorders (Ma et al., 1996; Miura et al., 1996). *Psoralea corylifolia* L. fruit preparations stimulated osteoblastic development in an *in vitro* grown UMR106 cell line, according to Wang et al. By using activity-guided fractionation, the flavonoids bavachin and corylin have been recovered and defined as bioactive components. These findings indicate that *Psoralea corylifolia* L. fruit preparations, as well as bavachin and corylin, may induce bone production or have anti-osteoporosis effect (Wang et al., 2001).

### *Epimedium davidii Franch* (Icariin, and Icaritin)

**FIGURE 2:**
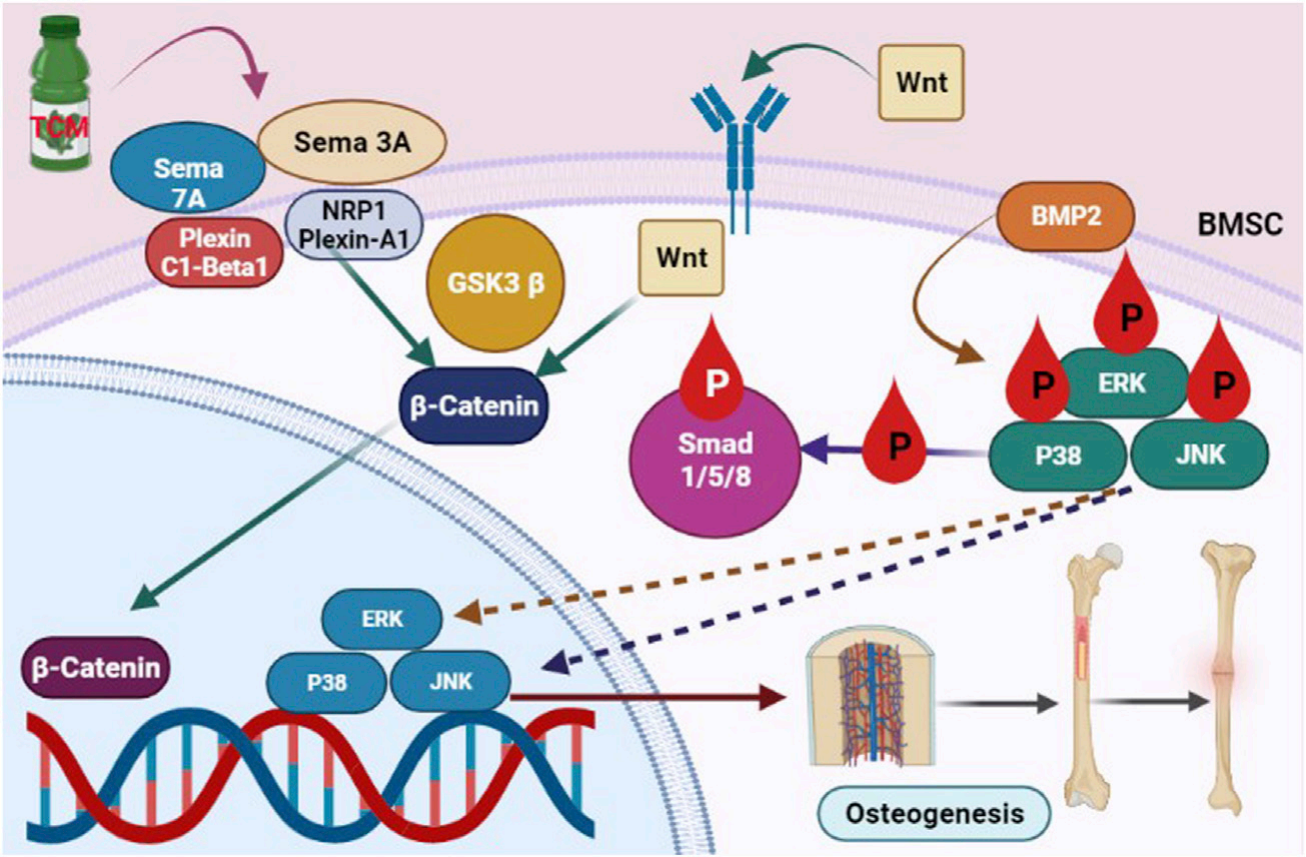
The Signaling pathway involved in TCM induced osteoblastogenesis.

*Epimedium davidii Franch* (EDF) was used to cure osteoporosis for decades, attributed to its ability to “develop” bones. Icariin is the major bioactive flavonoid glucoside extracted from EDFand it is linked to the herb’s pharmacological activities. Nevertheless, in current years, its bone-strengthening properties have gotten a lot of publicity. Icariin boosts antiosteoporotic action by promoting osteogenic development and calcification, decreasing adipogenesis, blocking osteoclast formation, and causing osteoclast death, all of which reduce osteoclast activity and bone resorption (Li et al., 2014b; Xu et al., 2016). Icariin may boost Nitric oxide (NO) production by increasing osteoblast development and matrix calcification. Subsequent research revealed that icariin therapy increased the expression of SMAD4, BMP-2, OPG, and Cbfa1/Runx2 genes while decreasing the expression of RANKL. This action might lead to its effects on osteoblast multiplication and development, which results in osteogenesis (Li et al., 2014b). Liang’s findings also showed that icariin might enhance osteogenesis in hFOB 1.19 cells *via* activating the BMP-2/Smad4 signaling cascade (Figure 2) (Liang et al., 2012). Icaritin, an icariin endogenous product, has a comparable performance to icariin. *In vitro* and in an OVX rat, oral administration of icaritin suppressed osteoclastogenesis by downregulating TRAF6 and inhibiting the NF-κB, MAPK/AP-1, and reactive oxygen species signalling cascades in a synchronized approach to diminish NFATc1 expression and activities (Tan et al., 2017). According to Wu and Sheng, icaritin increased the protein content of osteocalcin BMPs, and Runx2 in human adipose tissue-derived stem cells and derived mesenchymal stem cells while impeding adipogenesis in marrow mesenchymal stem cells by suppressing peroxisome proliferator-activated receptor gamma and glycogen synthase kinase-3 (Sheng et al., 2013).

TCM enhanced the Sema7A and sema3A-mediated signaling cascade, which is stimulated downstream of β-catenin nuclear translocation, and boosted the stimulation of the Smad1/5/8 and BMP2 pathway, leading to an increase in osteoblastogenesis.

### *Eclipta prostrata* (Mo-Han-Lian)

The aerial components of *Eclipta prostrata*L. (Asteraceae), often termed as “Mo-Han-Lian,” contain antiosteoporotic potential. Ecliptae herba produces the chemical wedelolactone. Though this Ecliptae herba ethyl acetate extract and wedelolactone had no effect on BMSC growth, the extract and wedelolactone did improve BMSC development into osteoblasts. The extract was given orally as well as applied topically on the fractured site. When BMSCs are incubated with wedeloloactone, the production of ALP, a diagnostic enzyme for mature osteoblasts, increases in a dose-dependent way. In addition, wedelolactone caused an elevation in bones calcification. Wedelolactone suppressed GSK3 function and increased GSK3 phosphorylation at the molecular scale, which therefore boosted nuclear translocation of β-catenin. The expression of genes associated to osteoblastogenesis, such as osteocalcin, osteorix, and runx2, was elevated. Wedelolactone treatment to ovariectomized rats reduced ovariectomy induced bone resorption by increasing osteoblast activation and stimulating osteogenesis (Zhang et al., 2008c; Zhang et al., 2013; Liu et al., 2016).

### Salvia miltiorrhiza

Researchers investigated the anti-osteoporotic potential of 99 distinct Chinese herbal remedies in 108 randomized studies involving 10,655 Chinese subjects. *Salvia miltiorrhiza* was present in 16 of the investigations. TCM therapy was contrasted to Western medication (like vitamin D2, caltrate, alendronate, and calcitonine) in 61 studies, including 23 natural treatment investigations showing a considerable benefit in boosting bone mineral density (BMD). In 48 researches the impact of combining Western medicines with Chinese herbal medicine, 26 found that the combined treatment had a greater effect on BMD versus Conventional medicine solo. The subsequent studies had equivalent results or were significantly lesser effective. Standard medicines such as denosumab, bisphosphonates, and selective oestrogen receptor stimulators have been often or not included in the controlled research utilizing Western medicine. Calcitriol caltrate, calcitonin, and vitamin D were the most often utilized Western medicines in the normal control in investigations including Salvia miltiorrhiza, whereas a bisphosphonate (alendronate) was given once only (Liu et al., 2014c).

## Network Pharmacology Approach for Active Ingredient Identification

Traditional Chinese medicine usually contain many components and the main issue of application of such combinations is that they contain the effective therapeutic components that have been identified or not yet and involve various unknown elements, some of which may have underlying toxicities. Therefore, it is extremely important to extract only these therapeutic components and avoid risks posed by the administration of raw herbal medicine. Therefore, there is a need to propose potent approaches to identify those therapeutic elements and extract them. In recent years, computer technology along with endless innovations and development in systems biology, a network pharmacology (NP) approach has been adopted which has shown applicational value in many fields, including drug target identification, discovery of active ingredients, investigation of mechanism of action, and safety evaluations (Ye et al., 2014). It was proposed for the first time in 2007 by Hopkins and is now believed as a promising approach which combine systems medicine with information science (Hopkins, 2007). It give an efficient approach for evaluation of the synergy of components of TCM and their mechanisms (Zhang et al., 2020) and has been widely used for the overall molecular mechanism of TCM preparations or multi-drug combinations (Ye et al., 2016). Bioinformatics tools are used to obtain differentially expressed genes (DEGs) as disease targets (e.g., Osteoporosis), and then effective components and pharmacological mechanism of TCM in the treatment of the disease are analyzed by network pharmacology. Various studies have reported this approach for the investigation of mechanism of TCM in treatment of Osteoporosis. For example, Li et al. used this approach for investigation of the mechanism of Xianlinggubao capsule in the treatment of osteoporosis (Li et al., 2021). They found that the active components (luteolin, quercetin, apigenin and ursolic acid) of Xianlinggubao capsule could be responsible for the mineralization of MC3T3-E1 cells. Similarly, Yang et al., used Network pharmacology approach combined with molecular docking to investigate the Potential Mechanism of Jintiange Capsule for the treatment of Osteoporosis and found that calcium phosphate (the main active ingredient), may interact with CALR and CALM1 targets and regulate multiple signaling pathways to treat osteoporosis (Yang et al., 2021). Gan et al. used NP approach for investigation of the mechanism of Rhizoma drynariae (TCM) against Osteoporosis and found 16 active ingredients that directly or indirectly target multiple signaling pathways and affect the differentiation and proliferation of multiple types of cells (Gan et al., 2019). There are many other examples where this approach has been used for identification of major components responsible for the treatment of osteophorosis and their possible mechanism has been provided. Yet, it is extremely important to extract only these therapeutic components and avoid risks posed by the administration of raw herbal medicine.

## Future Prospects

Accidental injuries and malignancies create bone abnormalities, which are widespread in clinical practice. High costs and long treatment cycles, uncontrolled curative impact, and problems such as infection, nonunion and malunion, of bones define existing bones defect therapies. These concerns not only have a negative impact on patients’ physiological and emotional health, but they also pose a difficulty for orthopaedic surgeons. The natural Chinese medications discussed in this article are both traditional and bone-specific. Therapeutic skills, as we all knew, are quite significant in Chinese medicine. As per the rich practises and expertise in the clinics, as well as Chinese medicine concepts, Chinese medications were grouped into several groups with specific functions. Many of them have been traditional and bone-specific medications used to manage bone injuries and bone—related illnesses because they improved bone production. In traditional Chinese medicine, the majority of them have the impacts and activities of tonifying the “Yang,” which improves bone formation and metabolism. In Chinese medicine, “yang-tonifying” medications are common and traditional type of natural remedies used to prevent osteoporosis (Ju et al., 2014; Li et al., 2015). Several clinical trials have demonstrated the antiosteoporosis actions of well substances, such as Epimedium derived phytoestrogen flavonoids, which were employed in a clinical study to cure and suppress osteoporosis and bone resorption in older women (Zhang et al., 2007; Zhu et al., 2012; Wang et al., 2013; Shi et al., 2017; Liang et al., 2020). TCM formulations offer less adverse effects, have become less expensive, and may be used for a prolonged time. TCM formulations not only can restore bones microarchitecture, enhance bone mass, and promote bone physiological qualities, but could also lessen or eradicate spinal debilitation, backache, as well as other complaints, according to a large body of clinical settings and animal experimentation (Zhu et al., 2012).

The modes of action of natural Chinese medicines that are efficacious in curing osteoporosis has not been thoroughly examined, emphasising the urgency for more research. To fully evaluate substances for pharmaceutical use, more studies are needed to identify and describe active antiosteoporotic molecules from traditional and bone-specific medications, as well as their safety, effectiveness, and probable interactions with other treatments. To establish their promising implications for the therapy of osteoporosis, as an efficient, potential substitute to principal treatment interventions, or in conjunction with existing main treatment modalities, research works to establish the special and focused molecular and cellular mechanisms of Traditional Chinese medicine substances are required.

## Conclusion

Countless trials have been performed to examine the pathophysiology of osteoporosis. Immunological modulation of osteoporosis is a new scientific focus that presents a novel method of osteoporosis etiology. The therapeutic potential of Traditional Chinese herbs in the management of osteoporosis has been demonstrated. Further investigation is necessary, but a growing number of researchers have shown that these herbs have an essential part in the rehabilitation of osteoporosis by modulating the immune system. Traditional Chinese medicine’s mode of action is notable for being multiroute and multitargeted, rather than a singular mechanism. Traditional Chinese medicine has therapeutic value for the management of osteoporosis, according to current *in vitro* and *in vivo* findings. To fully understand the therapeutic potential of Chinese medicines, more investigation is required to confirm their safety, potency, and accuracy. Further high excellence clinical trials using these traditional remedies are required to offer additional evidence for the candidate’s antiosteoporotic usage to be efficacious and non-toxic.

## Abbreviations

ACP, acid phosphatase; ACTH, Adrenocorticotropic hormone; BMD, bones mineral density; CCG, curculigoside; COG, Curculigo orchioides Gaertn; DXM, dexamethasone; EO, eucommia ulmoides olive; ERK, extracellular signal-regulated kinase; EX, Er-Xian decoction; FDA, Federal Drug Administration; FL, Fructus ligustri lucidi; GSK3-β, Glycogen Synthase Kinase 3-β; HE, Herba Epimedii; HRT, hormone replacement therapy; JNK, c-Jun N-terminal kinases; OPG, Orthopantomogram; OVX, ovariecto-mized; ROS, reactive oxygen species; TCM, Traditional Chinese medicines.
